# A genetic role for macrophage migration inhibitory factor (MIF) in adult-onset Still's disease

**DOI:** 10.1186/ar4239

**Published:** 2013-05-30

**Authors:** Fang-Fang Wang, Xin-Fang Huang, Nan Shen, Lin Leng, Richard Bucala, Shun-Le Chen, Liang-Jing Lu

**Affiliations:** 1 Department of Rheumatology, Renji Hospital, Shanghai Jiaotong University School of Medicine, 145 Middle Shan Dong Road, Shanghai, 200001, China; 2 Department of Medicine, Section of Rheumatology, Yale University School of Medicine, The Anlyan Center, 300 Cedar Street, New Haven, CT 06520-8031, USA

**Keywords:** macrophage migration inhibitory factor, adult onset still's disease, gene polymorphism, gene expression, DNA methylation

## Abstract

**Introduction:**

Adult-onset still's disease (AOSD) is a rare systemic inflammatory disorder in which abnormalities in inflammatory cytokines production appear to play a pathophysiological role. Our previous work has reported increased expression of macrophage migration inhibitory factor (MIF) and revealed its correlation with disease severity and activity in AOSD. A -173 G/C single nucleotide polymorphism (SNP) (*rs755622*) and a -794 CATT_5-8 _repeat (*rs5844572*) in the *MIF *promoter have been reported. In this study, we sought to explore the relationship between functional *MIF *promoter polymorphisms and MIF expression in AOSD.

**Methods:**

100 patients and 200 controls were recruited in the study. A polymerase chain reaction-restriction fragment length polymorphism (PCR-RFLP) assay was utilized to analyze the -173 G/C SNP (*rs755622*) and PCR-based size discrimination assay was applied to detect the -794 CATT_5-8 _repeat (*rs5844572*) in the *MIF *promoter. Plasma MIF levels were measured by ELISA. MIF mRNA levels were quantified by real-time reverse transcription (RT)-PCR. Bisulfate genomic sequencing was employed to evaluate DNA methylation status within the *MIF *promoter.

**Results:**

We identified that the frequencies of *MIF *-794 CATT_5 _(*P *= 0.001) allele and the expression of MIF (*P *<0.001) were increased in patients compared to healthy controls. Plasma levels of MIF in patients with CC genotype were higher than those of patients with GC or GG genotypes (*P *= 0.05). In patients with established AOSD, a higher frequency of -794 CATT_7 _containing *MIF *genotypes was observed in those with liver dysfunction (*P *= 0.009). Haplotype analysis revealed a higher representation of the *MIF *haplotype defined by -173*C/-794 CATT_5 _(C5) in AOSD patients (*P *= 0.001).

**Conclusion:**

Functional promoter polymorphisms in the *MIF *gene influence plasma MIF levels in AOSD and may contribute to disease susceptibility or clinical presentation of AOSD.

## Introduction

Adult-onset still's disease (AOSD) is a rare systemic inflammatory disorder characterized by spiking fevers, an evanescent salmon pink rash, arthritis, leucocytosis, and multiple organ involvement [1]. Although nosologically recognized for over one century, the etiology of this disease remains unknown. In a Canadian study, genetic evaluation revealed an association between HLA (human leukocyte antigen). B17, B18, B35, DR2 and AOSD [2]. Potential genetic risks for AOSD outside of the polymorphic HLA locus have also been explored. Sugiura and colleagues have reported that a promoter haplotype of the IL-18 gene was associated with AOSD in a Japanese population [3]. There is also evidence that monocyte/macrophage-derived inflammatory cytokines such as IL-1, IL-6, and TNF-α may play a physiological role in AOSD [4], which have been successfully therapeutically targeted in certain diseases [5,6].

Macrophage migration inhibitory factor (MIF) is a pleiotropic cytokine involved in the regulation of innate and adaptive immunity [7]. Its main actions are to counter-regulate the immunosuppressive and anti-inflammatory actions of glucocorticoids [8,9] and to inhibit the activation-induced apoptosis of macrophages [10]. The genetic deletion of MIF in animal models leads to a global decrease in the production of monocyte/macrophage-derived mediators such as IL-1β, IL-12, and TNF-α, confirming MIF's role as an upstream regulator of the inflammatory cascade [11], Evidence of a role for MIF during inflammation has been established in several autoimmune inflammatory diseases. Our previous work has revealed an association of serum MIF expression with disease activity and severity in AOSD [12], which was later confirmed by Becker and colleagues [13].

The molecular mechanisms regulating the expression of the MIF gene (*MIF*) [Genebank GeneID:307284] are still largely unknown. In recent years, genetic studies have established that functionally distinct alleles influence disease susceptibility and severity, and have supported prospects for pharmacological development. Functional polymorphisms in the *MIF *gene promoter have been studied in autoimmune diseases such as juvenile inflammatory arthritis [14], rheumatic arthritis [15], scleroderma [16], and systemic lupus erythematosus (SLE) [17]. A G/C single nucleotide polymorphism (SNP) at position -173(rs755622) [18] and a CATT tetranucleotide repeat at position -794(rs5844572) have been described and are associated with altered transcriptional activity of the *MIF *promoter. Based on these considerations, we sought to investigate a possible genetic link between *MIF *promoter polymorphisms and MIF expression in AOSD.

DNA methylation plays an important role in the epigenetic regulation of gene expression. Cytosine residues methylated by DNA methytransferases within CpG sites can inhibit gene transcription by interfering with the binding of the regulatory factors to DNA. A CpG island consisting of 34 CpG sites was detected in the *MIF *gene promoter (ranging from -300 base pairs to +1 base pair) and we further explored whether the methylation status of the *MIF *gene promoter affected *MIF *gene expression in AOSD.

## Materials and methods

### Recruitment of study subjects

A total of 100 patients (30 men and 70 women) who fulfilled the Yamaguchi criteria for AOSD were recruited into this study [19]. These patients were ethnic Han Chinese. Routine blood tests including erythrocyte sedimentation rate (ESR), serum ferritin, liver enzymes, rheumatoid factor (RF), antinuclear antibody (ANA), and anti-double-stranded DNA antibody were tested. Microbiology investigation, including bacterial and viral testing, imaging tests, and bone marrow and lymph node biopsies were performed when clinically indicated. Patients with infections, malignancies and other autoimmune diseases were excluded. Healthy volunteers biologically unrelated to patients were recruited as controls. Genomic DNA was extracted from all blood samples. All patients and controls were informed about the purpose of our study and consented to participate in the study. This study was approved by the institutional review board of Shanghai Jiaotong University.

### Polymerase chain reaction-restriction fragment length polymorphism (PCR-RFLP) genotyping for the *MIF *-173 G/C SNP

A 366-bp DNA fragment was produced by PCR with the forward primer 5'-ACT-AAG-AAA-GAC-CCG-AGG-C-3'and reverse primer 5'-GGG-GCA-CGT-TGG-TGT-TTA-C-3'. Amplification of 150ng of genomic DNA was performed in a 25-μL reaction volume containing 1.25 units of Taq DNA Polymerase, 1 μL of forward and reverse primer, 12.5 μL of 2× buffer and deoxynucleotide triphosphate (dNTP). The amplification consisted of an initial holding at 94°C for 3 minutes, followed by a three-step PCR program: 94°C for 30 seconds, 55°C for 30 seconds, and 72°C for 1 minute for 40 cycles. A final cycle of 72°C for 5 minutes completed the reaction. A 10-μL final reaction consisted of amplified PCR product (6 μL), 10× buffer (2 μL), FastDigest AIUΙ (1 μL) and distilled water (1 μL) was conducted at 37°C for 3 hours. The digested products were resolved on a 3% agarose gel and visualized using UV transillumination.

### Genotyping for the *MIF *-794 CATT_5-8 _repeat polymorphism

Genomic DNA (150 ng) was subjected to PCR for one cycle at 94°C for 3 minutes, 40 cycles at 94°C for 30 seconds, 55°C for 30 seconds and 72°C for 1 minute, followed by 72°C for 5 minutes. The forward primer was 5'-TTG-CAC-CTA-TCA-GAG-ACC-3' and was labeled with a FAM fluorescent dye, the reverse primer was 5'-TCC-ACT-AAT-GGT-AAA-CTC-G-3'. The 25 μL PCR reaction volume contained 150 ng of genomic DNA, 1.25 units of Taq DNA Polymerase, 1 μL of forward and reverse primer, 12.5 μL of 2× buffer and dNTP. The amplification included an initial holding at 94°C for 3 minutes, followed by a three-step PCR program: 94°C for 30 seconds, 55°C for 30 seconds and 72°C 1 minute for 40 cycles, and finally, 72°C for 5 minutes. The PCR product was appropriately diluted and supplemented with GeneScan 500 size standard (Applied Biosystems, Foster City, CA, USA) The mixture was then separated on a 50-cm polyacrylamide gel at 15000 V for 45 minutes on an ABI 3730XL capillary sequencer and analyzed with GeneMapper 4.0 software (Applied Biosystems). The four PCR product sizes were 207 bp, 211 bp, 215 bp,219 bp in length and these corresponded to 5-, 6-, 7-, and 8-CATT repeats respectively. To address the issue of multiple testing, we used the permutation test in R[20]. The permutation test was used in those instances in which the comparisons produced marginal *P*-values.

### MIF ELISA

Plasma MIF levels were measured using the quantikine human MIF kit (R&D systems, Minneapolis MN, USA). According to the suggested protocol, 100 μL of assay diluent and 50 μL of diluted standard or samples were added to a microplate precoated with capture antibody. After incubation for 2 hours at room temperature and four washes, 200 μL of MIF conjugate was added to each well for another 2 hours on the shaker. Color was developed by incubating with substrate solution for 30 minutes at room temperature. Stop solution was used to terminate the reaction and absorbance was read by a microplate reader set to 450 nm.

### Isolation of peripheral blood mononuclear cells and RNA preparation

Peripheral blood mononuclear cells **(**PBMC) were isolated from anticoagulated blood using Ficoll-HyPaque gradient centrifugation (Sigma-Aldrich, St Louis, Mo, USA), which was performed at 400 g for 30 minutes at 25°C. The PBMC-enriched interphase was collected and washed with PBS. Total RNA was extracted from PBMC using Trizol Reagent (Invitrogen, Carlsbad, CA, USA). The quality and quantity of total RNA were measured using a NanoDrop ND-1000 spectrophotometer (NanoDrop Technologies,Wilmington, DE, USA). Then RNA was puried using DNase as follows: 20 ìg of total RNA, 5 ìL of 10×DNase I buffer, 20 U of RNase inhibitor, 10 U of DNase I. The RNA then was quantified and up to 400 ng RNA was reverse-transcribed into cDNA in a final volume of 10 ìL using the Reverse Transcription Kit (Takara Bio Inc, Otsu, Japan).

### Real-time PCR

Expression levels of the *MIF *gene were quantified by real-time PCR with the SYBR Green PCR Master Mix (Takara). PCR reactions were carried out in triplicate with the 7900 Real-time PCR System (Applied Biosystems). The sequence of the primers used for PCR were as follows: forward primer: 5'-GAACCGCTCCTACAGCAAGCT-3'; reverse primer:

5'-GCGAAGGTGGAGTTGTTCCA-3'. The thermal cycling conditions included 10 minutes at 95°C and then 40 cycles of amplification for 3 seconds at 95°C and 20 seconds at 60°C. A melting curve analysis was performed after amplification. The efficiency of real-time PCR is 99.4%. The quantity of mRNA was calculated by normalizing the cycle threshold (CT) value of *MIF *to the CT of the housekeeping gene, glyceraldehyde-3-phosphate dehydrogenase (*GAPDH*), in the same sample according to the following formula: the average *GAPDH *CT was subtracted from the average *MIF *CT; the result represents the ΔCT. This ΔCT is specific and can be compared with the ΔCT of a calibration sample. The subtraction of control ΔCT from the ΔCT of thecase group is referred to as ΔΔCT. The relative quantification of expression of MIF was determined by using 2-ΔΔCT.

### Bisulfite methylation assay

Genomic DNA was extracted from PBMCs (Qiagen, Hilden, Germany) and 500 ng was bisulfite-modified using the EZ DNA methylation gold kit according to the manufacturer's instruction (Zymo research, Orange, CA, USA). We performed PCR of 250 ng of bisulfite-modified DNA for one cycle at 94°C for 3 minutes, 40 cycles at 94°C for 30 seconds, 55°C for 30 seconds and 72°C 1 minute, followed by 72°C for 5 minutes. The HotStart Taq DNA Polymerase system (Takara) was used: forward primer: 5'- TGTGGTTTAAAGATAGGAGGTATAGG -3'; reverse primer: 5'- TAATAACAAAAAAACCAAAAAACCC-3'. The PCR product was purified with a gel extraction kit and then cloned into the pGM-T vector. The positive cloning was selected for sequencing to confirm the methylation status of each CpG site within the *MIF *promoter (ranging from -300 base pairs to +1 base pair).

### Statistical analysis

The -173 G/C SNP and -794 CATT_5-8 _repeat genotype and allele frequencies in patients and controls were compared using Pearson's chi-square test and when appropriate, Fisher's exact test. No deviation from Hardy-Weinberg equilibrium was seen. The plasma MIF expression was presented as median ± SD. The non-parametric Mann-Whitney *U*-test was conducted to compare MIF expression between case-control groups or subset groups. All calculations were performed using SPSS version 13.0 analysis software. A permutation test was used. Given the multiple groups, the Bonferroni correction was applied and *P *<0.0167 was considered significant for the -173G/C data and *P *<0.0045 was considered significant for the -794CATT repeat data.

## Results

### Comparison of demographic and clinical characteristics of AOSD patients

The demographic and clinical characteristics of our recruited AOSD patients are shown in Table 1. There was a lower incidence of hydrohymenitis in our study group compared to other countries or ethnic populations. No apparent differences were found in the proportion of other clinical manifestations [21-24] (Table 1).

**Table 1:** Clinical manifestations of patients enrolled in the study

	This study (*n *= 100)	**Esdaile**[21] (*n *= 58)	**Larson**[22] (*n *= 17)	**Wouters**[23] (*n *= 45)	**Carreño**[24] (*n *= 20)
Male/female	3/7	3/4	10/7	2/3	9/11
Age, years, range	15, 70	16, 35	7, 44	16, 65	17, 57
Positive patients, %					
Fever(temperature ≥39°C)	99	83	100	84	100
Rash	88	90	84	82	80
Arthritis	98	98	100	98	100
Lymphadenopthy	47	45	48	36	50
Hepatoasplenomegaly	41	48	72	71	40
Hydrohymenitis	32	57	48	53	*

*Data incomplete.

### *MIF *-173 G/C SNP analysis

No deviation from Hardy-Weinberg equilibrium was observed in genotype frequencies for the polymorphisms. The -173G/C SNP genotype and allele

frequencies in AOSD patients and healthy controls are shown in Table 2. The CC genotype frequency was not significantly higher in patients than in controls (5% versus 0.5%, *P *= 0.017). There were no differences in the frequencies of the other two genotypes (GC *P *= 0.33; GG *P *= 0.072), and there was no difference in -173*C allele frequency between patients and controls (31% versus 21.5%, *P *= 0.072).

**Table 2:** Genotype and allele frequencies for -173 SNP

MIF -173 SNP	Patients (*n *= 100)	Controls (*n *= 200)	*P*-value
Genotype frequencies			
C/C	5(5%)	1(0.5%)	0.017
G/C	26(26%)	42(21%)	0.33
G/G	69(69%)	157(78.5%)	0.072
Allele			
-173*C	36(18%)	44(11%)	0.017

MIF, macrophage migration inhibitory factor; SNP, single nucleotide polymorphism.

### *MIF *-794 CATT_5-8 _repeat analysis

The -794 CATT repeat genotype and allele frequencies are shown in Table 3. The -794 CATT_5_-containing genotypes were significantly higher in the AOSD group compared with controls (49.5% versus 35.1%, *P *= 0.001) which suggests that the carriage of the -794 CATT_5 _allele may contribute to the development of AOSD. After permutation testing this difference remained significant (*P *= 0.018). There was no difference in the frequency of the high expression -794 CATT_7_-containing genotype in the comparison of AOSD patients with controls (17.4% versus 16.8%, *P *= 0.854). After permutation testing, the *P*-value was 0.911 in patients compared with controls.

**Table 3:** Genotype and allele frequencies for -794 CATT repeat polymorphism

MIF -794 STR	Patients (*n *= 95)	Controls (*n *= 188)	*P*-value
5/5	22(23.16%)	21 (11.17%)	0.008
5/6	34(35.79%)	75 (39.89)	0.503
5/7	15(15.79%)	14 (7.45%)	0.029
5/8	1(1.05%)	1 (0.53%)	0.621
6/6	9(9.47%)	34 (18.09%)	0.057
6/7	10(10.53%)	23 (12.23%)	0.673
6/8	0( 0%)	4 (2.13%)	0.152
7/7	4 (4.21%)	10 (5.32%)	0.685
7/8	0 (0%)	6 (3.19%)	0.078
Allele frequencies			
-794 CATT_5_	94(49.5%)	132(35.1%)	0.001
-794 CATT_7_	33(17.4%)	63(16.8)	0.854

MIF, macrophage migration inhibitory factor.

### *MIF *polymorphisms and AOSD clinical manifestations

AOSD patients were stratified according to clinical manifestations. The frequencies of the -174 G/C SNP and the -794 CATT_5-8 _genotypes were compared among patients with or without clinical features (myalgia, lymphadenopathy, liver damage, hydrohymenitis). Patients with liver damage, defined as elevated hepatic transaminases, had a higher frequency of the high-expression -794 CATT_7 _genotype compared with those without this manifestation(55.2% versus 30.8%, *P *= 0.009). No statistical differences were detected in other genotypes.

### Haplotype frequency analyses

The reconstructed frequencies of each possible haplotype based on the observed genotype data are shown in Table 4. The relative percentage of each haplotype was compared in AOSD patients and controls. The -173G/C and -794 CATT_5-8 _polymorphisms were in linkage disequilibrium. The AOSD patients had a significantly higher frequency of the haplotype defined by -173*C allele and -794 5-CATT repeats (C5) compared with controls (*P *= 0.001, odds ratio (OR) 5.534, 95% CI 1.712, 17.882) and the *P*-value on permutation testing was 0.021. The frequency of the G5 haplotype in AOSD patients also was higher than that in controls (*P *= 0.032, OR 1.474, 95% CI 1.034, 2.101). However, the *P*-value was 0.93 after permutation testing. There was a significant reduction in the -173*G/-794 6-CATT(G6) haplotype in AOSD patients (*P *= 0.007, OR 0.596, 95% CI 0.408, 0.871) and the *P*-value on permutation testing was 0.046.

**Table 4:** Comparison of haplotype frequencies in AOSD patients and controls

Haplotype	AOSD (%) *n *= 188	Control (%) *n *= 398	OR (95% CI)	*P*-value
C5	10(5.32)	4(1.00)	5.534 (1.712, 17.882)	0.001
C6	12(6.38)	28(7.04)	0.901 (0.448, 1.814)	0.770
C7	12(6.38)	21(5.28)	1.224 (0.589, 2.544)	0.588
G5	82(43.62)	138(34.67)	1.474 (1.034, 2.101)	0.032
G6	51(27.13)	153(38.44)	0.596 (0.408, 0.871)	0.007
G7	20(10.64)	42(10.55)	1.066 (0.612, 1.857)	0.822
G8	1(0.53)	12(3.02)	0.172 (0.022, 1.333)	0.057

AOSD, adult-onset Still's disease.

### Plasma levels of MIF

**Figure 1:**
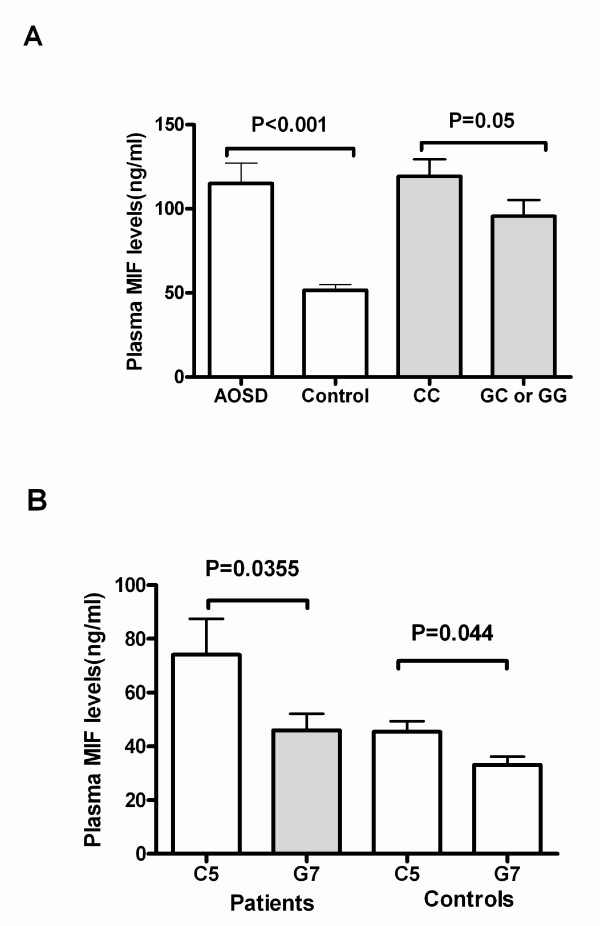
**Plasma levels of macrophage migration inhibitory factor**. (**A**) Comparison of plasma levels of macrophage migration inhibitory factor (MIF) between controls and patients and also in subsets of case group. (**B**) Comparison of plasma MIF expression in subgroups according to different haplotypes in both patients and healthy controls. AOSD, adult-onset Still's disease.

A difference in circulating plasma MIF levels was noted between AOSD patients and controls. The concentration of MIF was 83.28 ± 112.80 ng/mL in patients while 49.39 ± 29.27 ng/ml in controls (*P *<0.001, Figure 1A). Median concentrations of MIF were 110.7 ng/mL in the CC group and 76.43 ng/mL in the GC+GG group of patients (*P *= 0.05, Figure 1A). There was no difference between two groups stratified by the -794 CATT_5_-containing genotype or the -794 CATT_7_-containing genotype (not shown).

The association between plasma MIF levels and *MIF *haplotypes was explored. As shown in Figure 1B, patients with the C5 haplotype had higher serum MIF expression than those with the G7 haplotype (*P *= 0.0355). This was also observed in healthy controls (*P *= 0.044),

### DNA methylation status of the CpG island in the MIF promoter

**Figure 2:**
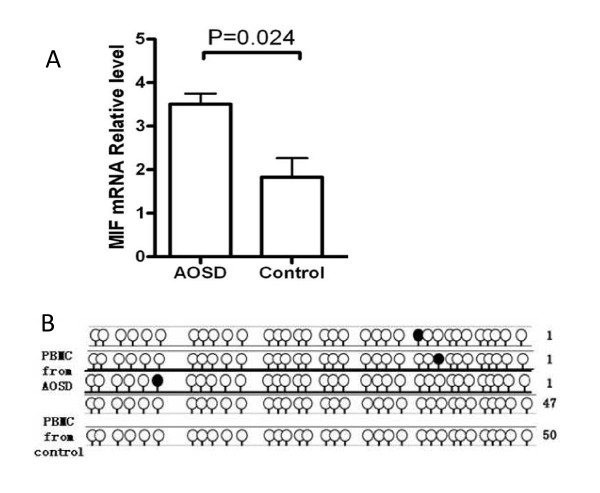
**Macrophage migration inhibitory factor (MIF) gene expression and DNA methylation status in *MIF *gene promoter in peripheral blood mononuclear cells (PBMC)**. (**A**) Real-time reverse transcriptase (RT)-PCR quantification of MIF mRNA relative level in the adult-onset Still's disease (AOSD) and healthy control groups. (**B**) Methylation status of 34 CpG sites of the proximal human MIF promoter in PBMC isolated from AOSD patients and healthy donors. Black circles represents methylated cytosines and white circles represents unmethylated sites.

The expression of MIF in PBMC was significantly increased in AOSD patients compared with controls (*P *= 0.017, Figure 2A). However, sequencing of PCR-amplified bisulfate-treated DNA indicated that only three methylated CpG sites were detected in three of fifty sequences from AOSD patients, while methylation was not detected in fifty sequences from ten healthy donors (Figure 2B).

## Discussion

AOSD is a systemic inflammatory disorder of unknown etiology. The incidence of this disease is relatively low and there are currently no reports of its prevalence in China. It is well-known that environmental factors and gene susceptibility play important roles in the pathogenesis of autoimmune inflammatory diseases. Genes involved in innate and adaptive immunity can contribute to immune abnormalities. MIF, an upstream regulator of cytokines involved in inflammatory diseases, is an excellent example. We previously showed serum MIF levels to be highly increased in AOSD patients compared with those with infection, neoplasm, and rheumatic arthritis, and in controls [13]. However, the factors leading to the abnormal production of MIF in AOSD remain unresolved. Based on our previous description of an association between MIF expression with severity and disease activity in AOSD and prior reports of a correlation between *MIF *functional promoter polymorphisms with several inflammatory diseases, we were prompted to study the role of *MIF *gene polymorphisms in disease susceptibility, clinical expression and plasma MIF concentrations in AOSD.

In the present study, we genotyped the -173 SNP and -794 CATT_5-8 _*MIF *promoter polymorphisms in AOSD patients and controls. The allele frequencies in control groups in our study were similar to another study in which Chinese Han were studied [25]. An increased frequency of haplotypes comprising -173*C/-794 CATT_5 _(C5) was observed in patients compared with controls, which confers an increased risk of AOSD. This partly differs from the previous finding that the *MIF *alleles, -173*C or -794 CATT_7_, are associated with the incidence or severity of a number of inflammatory conditions, including rheumatoid arthritis [15], juvenile inflammatory arthritis [16], inflammatory bowel disease [26], celiac disease [27] and asthma [28]. This association also was evident in a Spanish population diagnosed with SLE [29]. Notably, in a recent study of SLE, a dual effect of the -794 CATT polymorphism was noted with the low expression CATT_5 _found to be associated with disease susceptibility and the CATT_7 _allele with clinical severity [17]. Numerous studies based on defined reporter assays [15] or plasma MIF levels in different disease [17,30] support the designation of CATT_7 _as a high expression *MIF *allele. By contrast, there are fewer functional data for the -173*C allele, and its association with increased MIF expression may reside in its strong linkage disequilibrium with -794 CATT_7, _although to date, most studies have been confined to Caucasian populations. Notably, there are marked differences in the prevalence of -173*C in the Chinese versus Caucasian or African populations. In the present study, we found no significant difference in the frequencies of -173*C between patients and controls. However, Sreih and colleagues detected the frequency of -173*C-containing genotype was lower in SLE patients than in controls in African Americans but not in Caucasian populations [17]. It remains possible that this variant allele interacts functionally with the -794 CATT locus.

Subset analysis showed that plasma levels of MIF in AOSD patients with the CC genotype were higher than those with the GC or GG genotypes, although the difference was not significant. Our data also indicated that patients with established AOSD who had a -794 CATT_7 _containing genotype have a higher prevalence of liver damage. This conclusion is consistent with previous reports that *MIF *gene polymorphisms affect clinical manifestation and disease severity in certain inflammatory disorders [15,17,26,31].

Beyond the influence of promoter polymorphisms on the RNA transcription, DNA methylation is an additional, epigenetic mechanism that influences gene expression without affecting the underlying DNA sequence. We explored the association between the methylation status of a CpG island in the *MIF *promoter and MIF gene expression in PBMCs from AOSD patients and healthy donors. Our data indicate that DNA methylation is rare within the *MIF *promoter in both controls and AOSD patients. While these data suggest that DNA methylation may not be a significant mechanism affecting *MIF *expression in the PBMCs of AOSD patients, it remains possible that additional epigenetic mechanisms such as histone modification or small regulatory RNAs do play a role [32].

In summary, we investigated for the first time the association between functional *MIF *polymorphisms and AOSD. Our results suggest that *MIF *gene variants indeed play a role in susceptibility of AOSD, clinical manifestations, and plasma MIF expression. Further studies are in order to extend and confirm these initial findings in the Chinese Han as well as other populations and to validate the predictive value of *MIF *genotype or MIF plasma levels in the clinical course and treatment response.

## Conclusion

Functional promoter polymorphisms in the *MIF *gene influence plasma MIF levels in AOSD and may contribute to disease susceptibility or clinical presentation of AOSD.

## Abbreviations

ANA: antinuclear antibody; AOSD: adult-onset still's disease; bp: base pair; dNTP: deoxynucleotide triphosphate; CT: cycle threshold; ELISA: enzyme-linked immunosorbent assay; ESR: erythrocyte sedimentation rate; HLA: human leukocyte antigen; IL: interleukin; MIF: macrophage migration inhibitory factor; OR: odds ratio; PBMC: peripheral blood mononuclear cells; PBS: phosphate-buffered saline;PCR-RFLP: polymerase chain reaction-restriction fragment length polymorphism; RF: rheumatoid factor; RT: reverse transcription; SNP: single nucleotide polymorphism; TNF-α: tumor necrosis factor-α.

## Competing interests

The authors declare that they have no competing interests.

## Authors' contributions

All authors were involved in drafting the article. LJL had full access to all of the data in the study and takes responsibility for the integrity of the data and the accuracy of the data analysis. FFW was responsible for the collection of data. The analysis and interpretion of all data were finished by FFW, XFH, NS, LL, RB, SLC and LJL. All authors read and approved the final manuscript.
